# Reference values for the five-times-sit-to-stand test: a pooled analysis including 45,470 participants from 14 countries

**DOI:** 10.1007/s11357-025-01863-8

**Published:** 2025-08-28

**Authors:** Jozo Grgic, Brad J. Schoenfeld, Andrea B. Maier, Zeljko Pedisic

**Affiliations:** 1 NUS Academy for Healthy Longevity, Yong Loo Lin School of Medicine, National University of Singapore (NUS), Singapore, 117456 Singapore; 2 Department of Exercise Science and Recreation, Applied Muscle Development Lab, CUNY Lehman College, Bronx, NY USA; 3 Department of Human Movement Sciences, @AgeAmsterdam, Faculty of Behavioural and Movement Sciences, Amsterdam Movement Sciences, Vrije Universiteit Amsterdam, Van Der Boechorststraat 7, 1081 BT Amsterdam, The Netherlands; 4 Healthy Longevity Translational Research Programme, Yong Loo Lin School of Medicine, National University of Singapore, Singapore, 117597 Singapore; 5 School of Public Health, Li Ka Shing, Faculty of Medicine, The University of Hong Kong, Hong Kong, China

**Keywords:** Normative data, Norms, Sarcopenia, Physical performance, Longevity, Ageing, 5STS

## Abstract

The aim of this study was to establish reference values for the Five-Times-Sit-to-Stand Test (FTSST) in a large, population-representative sample from 14 European countries. Data were collected among 45,470 participants aged 50 + years, as part of the 5th wave of the Survey of Health, Ageing and Retirement in Europe. The reference values for the FTSST were calculated as the 5th, 10th, 20th, 30th, 40th, 50th, 60th, 70th, 80th, 90th, and the 95th weighted percentile. The analyses were stratified by age and sex. For females, the best performance on the FTSST was observed among the 50–54-year-olds (5th percentile = 18 s; 50th percentile = 10 s; 95th percentile = 6 s) and the worst among 85–89-year-olds and ≥ 90-year-olds (5th percentile = 28 s; 50th percentile = 15 s; 95th percentile = 9 s). For males, the best performance was observed among the 55–59-year-olds (5th percentile = 18 s; 50th percentile = 9 s; 95th percentile = 5 s). The worst performance for males was observed among ≥ 90-year-olds (5th percentile = 26 s; 50th percentile = 15 s; 95th percentile = 9 s), even though the 10th percentile was higher (poorer performance) among 85–89-year-olds. The reference values indicate poorer performance on the test among females and in older age groups. The provided reference values for FTSST can be used for health screening, surveillance, and intervention planning, as they enable an interpretation of test results according to sex and age.

## Introduction

The sit-to-stand tests are widely used to assess functional mobility and independence [1]. The tests are based on the amount of time needed to transition from sitting to standing [1]. There are several variants of the test, such as the Single Sit-to-Stand Test, Five-Times-Sit-to-Stand Test (FTSST), Ten-Times-Sit-to-Stand Test, and 30-Second Sit-to-Stand Test, with FTSST being the most commonly used [1–4]. The FTSST is designed for adults (18 + years of age). The test starts with the participant sitting on a straight-backed chair of standard height, and the task is to complete five sit-to-stand transitions, as quickly as possible. The time required to complete the task is recorded using a stopwatch.

The European Working Group on Sarcopenia in Older People recommends using either the handgrip strength test or the FTSST as the second step in diagnosing sarcopenia, following suspicion based on initial clinical examination or the SARC-F questionnaire (Strength, Assistance with walking, Rise from a chair, Climb stairs, and Falls) [5]. In case of poor performance on the handgrip strength test or FTSST, further screening for muscle mass quality or quantity (using dual-energy X-ray absorptiometry, bioelectrical impedance analysis, computed tomography, or magnetic resonance imaging) is recommended to confirm the diagnosis. Unlike the handgrip test that requires specialised equipment (i.e., a grip dynamometer), only a chair and a stopwatch are needed to conduct the FTSST highlighting its diagnostic utility, feasibility, and affordability. The FTSST is also used for other purposes, such as evaluating the efficacy of training interventions and the impact of nutritional supplements on exercise performance [6, 7]. This test is also included in the Short Physical Performance Battery, a common set of assessments used to evaluate functional status and physical performance [8]. The integrated care for older people (ICOPE) issued by the World Health Organization (WHO) also recommends the use of the FTSST to evaluate locomotor capacity [9].

A number of studies have been conducted on the measurement properties and utility of the FTSST. The test has adequate reliability and validity and it can discriminate between people with and without balance disorders [10, 11]. It can also be used to determine the risk of falls and to exclude the presence of moderate cognitive impairment [12–14]. Importantly, the test is safe, even among older and frail populations [15, 16].

To evaluate the performance of individuals, reference values for the FTSST have been established in different countries and population groups. For example, among (mostly) older adults in Colombia [17], India [18], Japan [19], Singapore [20] and Thailand [21], among middle-aged and older adults in Canada [22], and in all adult age groups (18 + years of age) in the Netherlands [23] and Portugal [24]. However, there is a lack of such studies in large multinational samples, especially those representing world regions (e.g., Asia, Europe, etc.). Such data would be valuable, as they would enhance generalizability across broader populations rather than being limited to those within a single country. While a meta-analysis conducted in 2006 summarised reference values for the FTSST from 14 studies [25], all but two of the included studies were from the US, the study was restricted to older adults, and only pooled means (not percentiles) for different age groups were reported.

Reference data are crucial for interpreting FTSST results, as they provide a benchmark for comparing an individual’s performance relative to their peers. Such data can help identify individuals with poor physical performance who may be at a higher risk of sarcopenia, balance disorders, falls, and cognitive impairment. Therefore, the aim of this study was to establish reference values for the FTSST in a large, population-representative sample from 14 European countries.

## Methods

### Study design and sample

Data were collected as part of the Survey of Health, Ageing and Retirement in Europe (SHARE), conducted in 28 European countries and Israel [26]. This survey has been conducted repeatedly since 2004 in population-representative samples of non-institutionalized adults aged 50 + years (primary responders) and their spouses. Our analysis used data collected in 2013 among 45,470 participants from countries in Europe as part of SHARE wave 5; the latest wave in which the FTSST was conducted. While the SHARE cohort includes participants from 29 countries, FTSST performance was evaluated in a subsample of 14 countries including Austria, Belgium, Czech Republic, Denmark, Estonia, France, Germany, Italy, Luxembourg, the Netherlands, Slovenia, Spain, Sweden, and Switzerland. All participants in the SHARE study provided informed consent, and the SHARE project has been reviewed and approved by the Ethics Council of the Max Planck Society as well as relevant national ethics committees in participating countries. A comprehensive description of the survey profile and procedures is provided elsewhere [26–31].

### Five-times-sit-to-stand test

Before starting the test, the participants were first asked to perform a single sit-to-stand transition. Only the participants who had been able to complete the trial were subsequently tested using FTSST, which was performed only once. The participants were asked to stand from a seated position and then sit, while keeping their arms folded across their chest, and to repeat this action five times as quickly as possible. The time needed for the participants to complete the task was measured using a stopwatch and expressed in seconds. The examiner gave instructions “Ready” and “Stand”, before starting the timer. The timing was stopped once the participant had fully stood after the final sit-to-stand transition. A lower recorded value indicated a better score on the test.

### Sociodemographic, health, and lifestyle characteristics

The following variables were used to describe the study sample: employment status (employed or self-employed, unemployed, retired, not working due to permanent sickness or disability, homemaker), place of residence (home or nursing home), self-reported health (excellent, very good, good, fair, or poor), limitations in the five activities of daily living (scored 0 to 5), mobility limitations (scored 0 to 7), body mass index (< 18.5, 18.5–24.9, 25.0–29.9, > 30 kg/m^2^), smoking status (non-smoker or current smoker), and frequency of participation in moderate and vigorous physical activity (> 1 time per week, once per week, 1–3 times per month, hardly ever or never).

### Statistical analysis

Absolute frequencies and weighted percentages were calculated for all sociodemographic, health, and lifestyle categories. The reference values for the FTSST were calculated as the 5th, 10th, 20th, 30th, 40th, 50th, 60th, 70th, 80th, 90th, and the 95th weighted percentile, where a higher percentile indicates better performance on the test (e.g., a score on the 95th percentile indicates that the given individual scored equal or better than 95% of people in the respective population group). The percentiles were calculated separately for the following age groups: 50–54, 55–59, 60–64, 65–69, 70–74, 75–79, 80–84, 85–89, and 90 + years of age. The percentiles were also calculated for middle-aged adults (50–64 years of age), older adults (65 + years of age), and the whole sample (50 + years of age). All the analyses were stratified by sex. The sample weights (person-level analysis weight) provided in the SHARE dataset were used to improve the generalizability of results. All analyses were performed using IBM SPSS Statistics version 30 (SPSS Inc. an IBM Company, Chicago, IL, USA).

## Results

### Participant characteristics

There were 54% of females in the study sample (Table 1). The sample was split almost evenly between middle-aged (51.1%) and older adults (48.9%). The most represented group according to the employment status were retirees (44.8% among females and 51.9% among males). Almost all participants (> 99.5%) lived at home at the time of the study. More than 70% of participants reported good, very good or excellent health, and ~ 95% of the sample did not require assistance with activities of daily living. Prevalence of no mobility limitations was higher among male participants (70.5%) than among female participants (55.7%). Between 31.9% and 45.3% of participants had a body mass index between 18.5 and 24.9 kg/m^2^, and 77.1–82.9% of the study sample were non-smokers. Nearly three out of four participants (72.6–74.4%) reported engaging in moderate physical activity more than once per week, while such regular participation in vigorous physical activity was less prevalent (34.0% among females and 43.2% among males).

**Table 1:** Characteristics of female (*n* = 24,562) and male (*n* = 20,908) participants

Characteristic	Frequency (weighted percentage)	
	Females	Males
*Age (years)*		
50–54	3,501 (50.4)	2,467 (49.6)
55–59	4,749 (50.5)	3,643 (49.5)
60–64	4,732 (50.8)	4,116 (49.2)
65–69	4,300 (50.6)	3,938 (49.4)
70–74	3,220 (51.2)	3,015 (48.8)
75–79	2,224 (53.7)	2,049 (46.3)
80–84	1,252 (56.9)	1,185 (43.1)
85–89	478 (59.9)	398 (40.1)
90 +	106 (58.4)	97 (41.6)
*Employment status*		
Employed or self-employed	7,349 (31.8)	6,913 (38.9)
Unemployed	653 (3.3)	725 (5.7)
Retired	12,384 (44.8)	12,518 (51.9)
Not working due to permanent sickness or disability	612 (2.1)	494 (2.3)
Homemaker	3,186 (16.3)	51 (0.3)
Other	281 (1.3)	151 (0.7)
*Residence*		
Home	24,464 (99.6)	20,855 (99.7)
Nursing home	83 (0.3)	46 (0.3)
*Self-reported health*		
Excellent	2,415 (8.9)	2,080 (9.2)
Very good	4,846 (18.2)	4,254 (19.0)
Good	9,978 (41.9)	8,634 (43.0)
Fair	6,156 (25.7)	4,927 (23.7)
Poor	1,160 (5.3)	1,006 (5.1)
*Activities of daily living score*^a^		
0	23,209 (94.1)	19,851 (95.1)
1	991 (4.3)	781 (3.6)
2	244 (1.0)	179 (0.8)
3	67 (0.2)	46 (0.1)
4	18 (0.1)	22 (0.1)
5	28 (0.2)	29 (0.2)
*Mobility score*^b^		
0	13,779 (55.7)	14,533 (70.5)
1	4,560 (17.7)	3,320 (15.2)
2	2,880 (11.9)	1,663 (7.6)
3	1,748 (7.3)	768 (3.9)
4	943 (4.2)	361 (1.6)
5	436 (2.1)	161 (0.7)
6	155 (0.8)	62 (0.2)
7	59 (0.3)	39 (0.3)
*Body mass index (kg/m*^*2*^*)*		
< 18.5	411 (1.9)	75 (0.4)
18.5–24.9	10,435 (45.3)	6,598 (31.9)
25.0–29.9	8,525 (32.9)	10,066 (48.9)
> 30	4,681 (17.7)	3,991 (18.0)
*Smoking status*		
Non-smoker	20,546 (82.9)	16,608 (77.1)
Current smoker	4,015 (17.1)	4,299 (22.9)
*Moderate physical activity*		
> 1 time per week	18,629 (72.6)	16,027 (74.4)
1 time per week	3,211 (13.6)	2,776 (14.3)
1–3 times per month	1,230 (5.8)	1,051 (5.5)
Hardly ever or never	1,487 (8.1)	1,051 (5.8)
*Vigorous physical activity*		
> 1 time per week	8,961 (34.0)	9,199 (43.2)
1 time per week	3,942 (14.8)	3,189 (14.4)
1–3 times per month	2,205 (8.1)	1,984 (9.1)
Hardly ever or never	9,445 (43.0)	6,532 (33.3)

^a^A lower score indicates a higher level of functional independence

^b^A lower score indicates better mobility

### Reference values for the five-times-sit-to-stand test

For females, the best performance on the FTSST was observed among the 50–54-year-olds (5th percentile = 18 s; 50th percentile = 10 s; 95th percentile = 6 s) and the worst among 85–89-year-olds and ≥ 90-year-olds (5th percentile = 28 s; 50th percentile = 15 s; 95th percentile = 9 s; Table 2). For males, the best performance was observed among the 55–59-year-olds (5th percentile = 18 s; 50th percentile = 9 s; 95th percentile = 5 s; Table 3). The worst performance for males was observed among ≥ 90-year-olds (5th percentile = 26 s; 50th percentile = 15 s; 95th percentile = 9 s), even though the 10th percentile was higher (poorer performance) among 85–89-year-olds. Compared with females, males had better scores in 70, equal scores in 24, and worse scores in 5 out of 99 percentile-by-age comparisons, with the maximum difference of −5 s in favour of males and −2 s in favour of females.

**Table 2:** Reference values for the Five-Times-Sit-to-Stand Test for females (pooled *n* = 24,562)

Age (years)	Weighted percentile (seconds)^a^										
	5th	10th	20th	30th	40th	50th	60th	70th	80th	90th	95th
50–54	18	15	12	11	10	10	9	8	7	6	6
55–59	20	16	13	12	11	10	9	9	8	7	6
60–64	20	17	14	12	11	10	10	9	8	7	6
65–69	22	18	15	13	12	11	10	9	9	8	7
70–74	20	18	15	14	13	12	11	10	9	8	7
75–79	23	20	17	15	14	12	12	10	9	8	7
80–84	25	21	18	16	15	13	13	12	11	9	8
85–89	28	24	20	18	17	15	14	13	12	10	9
90 + ^b^	28	25	21	20	17	15	13	12	12	9	7
50–64	20	16	13	12	11	10	9	8	8	7	6
65 + ^b^	23	20	16	15	13	12	11	10	9	8	7
50 + ^b^	21	18	15	13	12	11	10	9	8	7	6

^a^A lower value indicates a better score on the test

^b^The oldest participant was 100 years old

**Table 3:** Reference values for the Five-Times-Sit-to-Stand Test for males (*n* = 20,908)

Age (years)	Weighted percentile (seconds)^a^										
	5th	10th	20th	30th	40th	50th	60th	70th	80th	90th	95th
50–54	19	15	12	11	10	9	9	8	7	6	5
55–59	18	15	12	11	10	9	9	8	7	6	5
60–64	18	15	13	12	11	10	9	8	8	6	6
65–69	20	16	14	12	11	10	9	9	8	7	6
70–74	20	17	15	13	12	11	10	9	8	7	6
75–79	22	18	15	14	12	12	10	10	9	8	7
80–84	23	20	17	15	14	13	12	11	10	8	8
85–89	25	23	18	15	14	13	12	10	10	8	7
90 + ^b^	26	20	18	17	16	15	14	13	11	10	9
50–64	18	15	12	11	10	9	9	8	7	6	5
65 + ^b^	21	18	15	13	12	11	10	9	8	7	6
50 + ^b^	20	16	14	12	11	10	9	8	8	7	6

^a^A lower value indicates a better score on the test

^b^The oldest participant was 98 years old

Reference values for middle-aged adults (50–64 years of age) and older adults (65 + years of age) followed a similar pattern. Better performance across all percentiles was observed for middle-aged adults. These age-related differences ranged from −1 to −4 s for females and from −1 to −3 s for males. Compared with females, males had better scores in 20 and equal scores in 2 out of 22 percentile-by-age comparisons.

## Discussion

### Key findings

This study provided reference values for the FTSST, established in a large multinational sample. The reference values indicate poorer performance on the test among females and in older age groups. The established FTSST reference data can serve as a benchmark, allowing clinicians to interpret functional mobility and independence of individuals relative to their peers.

### Comparison with previous studies

Compared with reference values provided in a study from Colombia [17], most of the reference values in our study were 2–4 s lower for females and 1–3 s lower for males. Compared with reference values provided in a study from Canada [22], most of the reference values in our study were 1–2 s lower for females and 1–3 s lower for males, except for the 5th and 10th percentiles which were mostly equal to or higher than in the Canadian study. These discrepancies in reference values may be due to differences in test protocols. Prior to FTSST, participants in the Colombian and Canadian studies were involved in several lower-body tests (e.g., gait speed, different balance tests, and/or timed-up-and-go), while the participants in our study performed a handgrip test (upper-body strength) only [17, 22]. Therefore, fatigue may have influenced performance on the FTSST in the Colombian and Canadian studies, which would explain better test performance observed in our study.

Compared with the medians from a Portuguese study [24], the 50th percentiles for females in different age groups in our study were generally 1–2 s higher, while no clear pattern of differences could be seen for the respective reference values among males. These differences might be due to the different number of repetitions of the test. While the participants in the Portuguese study repeated FTSST three times, our study included only one repetition of the test. This may have affected the results, because it is likely that participants in the Portuguese study became more accustomed to the testing procedure compared to participants in our study.

The reference values in our study were considerably higher when compared with the values from a Japanese study [19]. However, this was not unexpected, because such large discrepancies of Japanese results in the FTSST, compared with results from Western countries, have previously been reported in Bohannon’s review paper [25]. Some of these differences could be due to Japanese lifestyle, which includes the frequent use of squatting movements, tatami mats, and futon bedding [32]. 

The percentiles in our study were generally 3–4 s lower than in a study from Thailand [21]. However, the Thai study used a slightly different measurement protocol, with the test ending with the participant in the seated position. The additional time needed to sit after the fifth rise into the standing position is the reason why this test variant is proposed to yield slightly higher times. Interestingly, the 50th percentiles for different age groups in our study were very similar to the pooled means in a 2006 review paper [25], even though they combined results from studies using both variants of the test ending (i.e., in the seated or standing position).

While our study was the first to determine European reference values for the FTSST, a similar analysis was recently conducted for Asia, using a pooled sample from Japan, Malaysia, and Taiwan [33]. In that study, across the age groups from 50–54 to 80 + years (oldest age group in the study), the 5th, 50th, and 95th percentiles ranged from 13–15, 8–9, and 4–6 s for males, and from 14–17, 8–10, and 5–6 s for females, respectively. These values are generally lower than those reported in our study, suggesting that the Asian population may perform better on the FTSST, compared with the European population. Among others, this study also pooled data from Japan, which is important to consider when interpreting these differences, given the previously mentioned high performance of Japanese in the FTSST [19, 25, 32]. The previous study also established cut-offs for poor performance, but it is important to note that these focus on sarcopenia, while different cut-offs may be needed for other outcomes. Although no universally accepted cut-off exists, the 20th percentile is commonly used for this purpose [33, 34].

Overall, the better performance on the FTSST among males observed in our study aligns with findings from previous research [17, 18, 21–23], although some studies did not find such differences [24]. The age-related decline in performance on the test also follows the pattern established in previous studies [17, 19, 21–25]. It is interesting that the 60th – 95th percentiles on the FTSST were slightly lower (indicating better performance) among the oldest females in our sample (90 + years of age), compared with the second oldest category (85–89 years of age). A possible reason for this could be that greater muscle strength is associated with higher life expectancy [35].

### Practical applications

From a practical perspective, the reference values for the FTSST can be used for health screening, surveillance, and intervention planning. These data can help identify individuals with below-average performance, which may indicate risk for falls, frailty, or mobility limitations [10, 11]. They can also be used as an early indicator of decline in performance and to prescribe interventions (e.g., exercise, dietary supplements) [36]. On population-level, the reference data can be used to evaluate trends in performance across different demographic groups over time.

### Strengths and limitations

A key strength of the current study is that the analyses were conducted in a large, population-representative sample from 14 countries. When interpreting the reference values for the FTSST, it is important to consider that the SHARE cohort in each of the included countries is nationally representative. As such, each national study sample reflects a broad spectrum of various characteristics that may be relevant to the FTSST (e.g., various levels of self-reported health, mobility, and physical activity). Therefore, the reference values are applicable to the entire population, including the members of specific population groups (e.g., individuals with low self-rated health, mobility, and physical activity level). Members of a specific population group may have lower or higher scores in the FTSST compared with other population groups, and this will be reflected when comparing their scores with the reference values. Another key strength of this study was the application of the FTSST without previously involving participants in other tests that could cause fatigue in the legs.

There are also some limitations of the study that need to be considered. First, the chair height used for the FTSST was not specified in the test protocol. Chair height can influence performance on this test, and while heights of 43–47 cm are typically used [37], it remains unclear whether this was consistently applied across all assessments in the current study. Second, the data were collected in 2013. However, a study from Portugal found no temporal variation in a similar sit-to-stand test between 2008 and 2018, suggesting that our findings are likely to remain generalizable over time [38]. Still, researchers managing the SHARE cohort could consider re-introducing the FTSST in future waves of data collection, to allow analyses of trends in performance. Additionally, the data were collected in the EU, which may limit the generalisability of our findings to predominately European population, highlighting that future studies may be needed to established reference values for other populations.

## Conclusion

This study established reference values for the FTSST in a large multinational sample. The reference values indicate poorer performance on the test among females, compared with males, and in older age groups, compared with younger age groups. The reference values for the FTSST can be used for health screening, surveillance, and intervention planning, as they enable an interpretation of test results according to sex and age.

## Data Availability

Data used for this study are available upon request from the Survey of Health, Ageing and Retirement in Europe (SHARE) website: https://share-eric.eu/.
